# Phthisis

**Published:** 1889-11-16

**Authors:** 


### The Practitioner's Outlook and Retrospect.

[*The Editor will be glad to receive offers of co-operation and contributions from members of the profession. There is no doubt that much valuable material full of interest and instruction is at present lost to science. This is largely so in the case of* (1) *the younger and less-known members of the hospital staff,* (2) *those connected with cottage hospitals, and* (3) *the great body of medical practitioners not attached to any institution. The co-operation of all is cordially invited to enable the Editor to make* The Hospital *of the utmost possible use and value to the profession. Specialists willing to review books on their special subjects are invited to communicate this fact at once. All letters should be addressed to* The Editor, The Lodge, Porchester Square, London, W.]

### MEDICINE.

### PHTHISIS.

Dr. W. H. Pearse, senior physician to the Plymouth Dispensary, gives some interesting illustrations of consumption in the *Provincial Medical Journal* for October, 1889. In one family six children died of phthisis, although no phthisis could be traced in the family for three generations. But, as Dr. Pearse observes, "not the less can heredity be excluded, for we are the children of a thousand and one generations." But in other respects this family exhibited atavism and reversion both in function and structure. All the females had scant menses or complete amenorrhœa. Four of the children had a peculiarity of the index finger nail as possessed by the mother. As regards treatment Dr. Pearse lays great stress on environment, and mentions that he has seen a number of young people who had symptoms of prœ-phthisis, or even slight physical signs of phthisis get well from a month or two of change of air. What Darwin says on the vast power of good or evil on plants or animals—even determining their sterility or fecundity—of slight changes of environment is equally true of man. An usual prescription of Dr. Pearse's is quinine and sulphuric acid, and in many cases he has combined arsenic usefully. We agree, however, with the author that the main requirement in the treatment of phthisis is change of air, but the change must be made with direct reference to the stage of the malady, the climate benefiting one stage not usually suiting another.

